# Altitude-adaptive water use strategies of grassland are constrained by air dryness and stoichiometry in southwest of China

**DOI:** 10.3389/fpls.2026.1773262

**Published:** 2026-02-09

**Authors:** Jiankun Bai, Deping Zhai, Yang Xu, Deyun Chen, Wei Wang, Ziyue Xu, Yuhui Si, Fujia Yang, Mei Sun, Yinfeng Zhang, Zhigang Chen, Juan Yang, Wenhui Cui, Junbao Yu, Xiaoli Cheng

**Affiliations:** 1 Yunnan Key Laboratory of Plateau Wetland Conservation, Restoration and Ecological Services, College of Ecology and Environment, Southwest Forestry University, Kunming, China; 2 National Plateau Wetlands Research Center, Southwest Forestry University, Kunming, China; 3 School of Ecology and Environmental Sciences, Yunnan University, Kunming, China; 4 State Key Laboratory of Vegetation Structure, Function and Construction, Yunnan University, Kunming, China

**Keywords:** atmospheric conditions, climate change, elevation gradient, grassland, stable isotope, stoichiometry, water use strategies

## Abstract

**Introduction:**

Understanding the elevational patterns of intrinsic water-use efficiency (iWUE) and their drivers is crucial for predicting plant adaptation and ecosystem responses to climate change. However, how iWUE in different photosynthetic pathways (C_3_ vs C_4_) varies with elevation, which is interactively shaped by climate and nutrient constraints remains unclear.

**Methods:**

Here, we integrated stable carbon (δ^13^C) and oxygen (δ^18^O) isotopes with plant-soil stoichiometry across a grassland elevation transect to interpret these mechanisms.

**Results:**

Our results reveal a fundamental divergence in the response of iWUE to elevation: iWUE increased significantly in C₃ grasses but decreased slightly in C_4_ grasses. Using a machine learning approach, we identified vapor pressure deficit (VPD) and leaf stoichiometry (C:P and N:P ratios) as key drivers to shape the altitudinal patterns of iWUE. However, these factors exhibited opposing effects: VPD was negatively correlated with iWUE in C_3_ species but positively correlated in C_4_ species.

**Discussion:**

These contrasting patterns reflect distinct eco-physiological strategies. C_3_ plants improve iWUE under the cooler, potentially nutrient-limited in high-elevation conditions through conservative resource-use traits. In contrast, the CO_2_-concentrating mechanism of C_4_ plants appears constrained at lower temperatures, limiting their iWUE. Our findings demonstrate that iWUE patterns are not simply climate-driven but emerge from pathway-specific interactions between climatic gradients and nutrient availabilities. This study provides a mechanistic framework for forecasting shifts in grassland community structure and carbon-water fluxes under future climate change.

## 1 Introduction

Water use efficiency (WUE), defined as the ratio of carbon assimilation to water loss, serves as a fundamental metric for evaluating the resilience of terrestrial ecosystems to climate change (Franks et al., 2015; Li et al., 2023). In terrestrial ecosystem, understanding how plants balance carbon gain against water loss is critical for predicting future carbon sequestration and hydrological feedbacks (Farooq et al., 2019; Migliavacca et al., 2021). Traditional methods for measuring WUE, such as gas exchange measurements, are often limited in temporal and spatial scalability (Franks et al., 2015). The analysis of stable carbon isotope composition (δ¹³C) in plant tissues has emerged as a powerful, integrative tool to quantify intrinsic water use efficiency (iWUE) over the time scale of plant growth, as δ¹³C discrimination during photosynthesis is linked to the interplay between stomatal conductance (*g_s_*) and photosynthetic capacity (*A*) (Maxwell et al., 2018; Rumman et al., 2018). This dual-isotope approach provides a novel tool to decode plant eco-physiological strategies (Eitelberg et al., 2025). To robustly generalize the patterns and drivers underlying the variation of δ¹³C, investigations spanning extensive natural environmental gradients are essential. Altitudinal transects, which provide dramatic and co-varying gradients in temperature, moisture, and atmospheric pressure over relatively short distances, serve as a unique and powerful “natural laboratory” for exploring how plant functional traits and adaptive strategies respond to multivariate environmental changes (Körner, 2007).

The iWUE of plants is fundamentally shaped by the interaction between stomatal conductance and photosynthetic capacity, both of which are highly sensitive to climatic drivers (Brümmer et al., 2012; Huang et al., 2016). Among these factors, vapor pressure deficit (VPD) is a key driver of stomatal behavior (Yuan et al., 2025). As air dryness increases with elevation rising, the gradient driving water vapor diffusion from leaves to the atmosphere intensifies, potentially leading to stomatal closure to prevent excessive water loss (Mathias and Thomas, 2021; Li et al., 2023). This phenomenon has been observed in various grass species, where elevated VPD conditions during growth periods were found to increase bundle-sheath leakiness and intrinsic water use efficiency (Guerrieri et al., 2019; Hatfield and Dold, 2019). Temperature is another critical climatic factor that controls WUE through its direct effects on metabolic rates and stomatal kinetics (Sage and Kubien, 2007; Koehler et al., 2010). Rising temperatures generally accelerate leaf respiration and transpiration rates, which can reduce WUE if photosynthesis does not keep pace (Hatfield and Dold, 2019; Xu et al., 2023). In mountainous regions, the combined effects of temperature and VPD create a dynamic environment where plants must rapidly adjust their stomatal aperture to maintain optimal hydration while maximizing carbon uptake (Liu et al., 2022; Bai et al., 2025). Nevertheless, the counteracting effects of increasing VPD in response to global warming poses a significant threat to the long-term stability of iWUE in many terrestrial biomes (Zhan et al., 2025). Therefore, understanding how these climatic variables interact to regulate iWUE is key to accurately modeling carbon-water coupling in grassland ecosystems.

Beyond climatic drivers, the internal physiological traits of plants, as reflected by their nutrient’s composition (plant stoichiometry), plays a crucial role in regulating iWUE (Cernusak et al., 2010; Yan et al., 2016). The allocation of nutrients such as nitrogen (N) and phosphorus (P) directly affects the activity of photosynthetic enzymes and the structure of leaf tissues, thereby influencing the iWUE (El-Madany et al., 2021). For instance, nitrogen content is closely linked to the maximum rate of carboxylation (*V_cmax_*) and the capacity for Rubisco regeneration, both of which are key determinants of photosynthetic efficiency and stomatal conductance (Dawson et al., 2022). In grasslands, the nutrients availability often dictates the competitive ability of different functional groups, influencing their dominance along altitudinal gradients and consequently shaping the overall ecosystem iWUE (Bai et al., 2025; Karitter et al., 2025). Moreover, soil properties also constrain iWUE by regulating root water uptake and the hydraulic conductivity of the soil-plant-atmosphere continuum (SPAC) (Orlowski et al., 2023). Soil texture, organic matter content, and water holding capacity determine the availability of soil moisture, which acts as an important limiting factor for stomatal behaviors (Sun et al., 2023). Thus, a comprehensive understanding of iWUE should integrate both the external climatic drivers and the internal physiological constraints imposed by plant stoichiometry and soil properties.

Altitude gradients serve as a natural laboratory, where temperature, precipitation, and VPD vary systematically over short distances, which offers a unique way to study the adaptive strategies of grassland plants (Wu et al., 2015; Midolo et al., 2019). In particular, the study of iWUE along altitudinal gradients provides critical insights into the evolutionary and physiological mechanisms that enable plants to survive in extreme environments. Therefore, integrating altitudinal studies with stable isotope analysis provides a powerful approach to reveal the complex interactions between plant physiology, soil properties, and climate drivers that govern iWUE in terrestrial ecosystems. Thus, our study was aim to address two questions: First, how does the iWUE of different photosynthetic pathways (C_3_ vs C_4_) vary along an elevation gradient? Second, how do the integrated effects of climatic, plant, and soil factors influence these altitudinal patterns of iWUE in grasslands?

## 2 Materials and methods

### 2.1 Field survey and sampling

**Figure 1:**
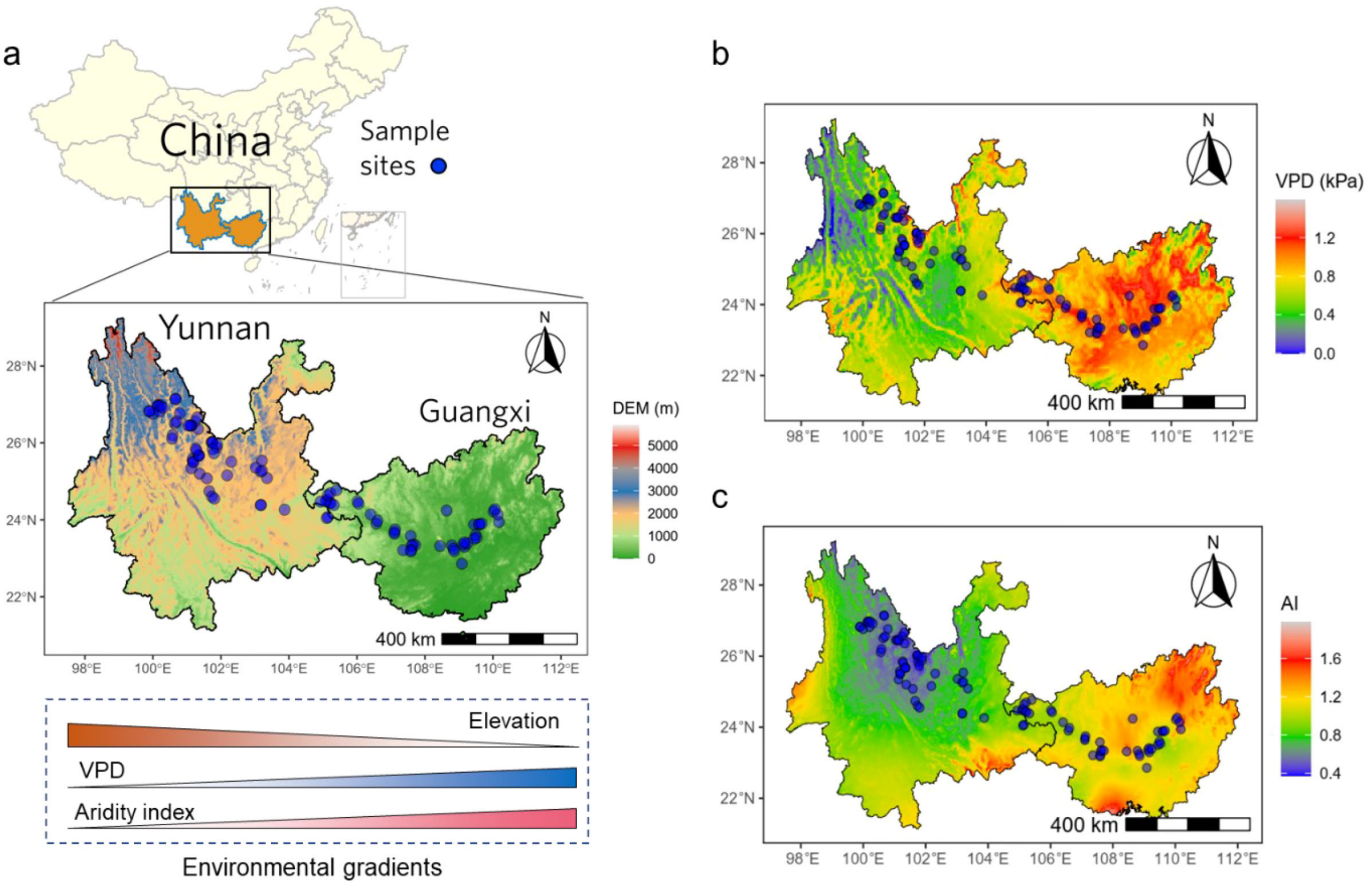
The geographical and environmental gradients of sampling locations in the grasslands of southwestern China. The locations of sampling sites were selected along an elevational gradient **(a)**, vapor pressure deficit (VPD) **(b)**, and aridity index (AI) **(c)**.

A grassland transect was established along an elevational gradient in southwestern China, extending approximately 1,000 km between 40 and 3800 m above sea level (**Figure 1**). This region is globally recognized as a biodiversity hotspot, characterized by verticalenvironmental gradients and complex topography across elevations, which promote highly heterogeneous vegetation patterns (Rahbek et al., 2019). During the 2021 growing season (July and August), 98 sampling sites were surveyed along the transect. At each location, a main plot of 30 m × 30 m was set. Within it, six 1 m × 1 m subplots were typically positioned at the plot center and each corner. In cases where grassland cover was limited (area< 30 m × 30 m), only three subplots were surveyed. All vascular plant species within each subplot were identified and their abundances recorded. One to three dominant species per site were selected, and a minimum of three replicates per species were collected for further analysis. Plant species were categorized as C_3_ or C_4_ based on isotopic analysis conducted with an isotope ratio mass spectrometer. Across the transect, mean annual temperature (MAT) varied from 7.6 to 23.6 °C, mean annual precipitation (MAP) ranged between 730 and 1760 mm, vapor pressure deficit (VPD) varied from 0.2 to 1.3 kPa, while aridity index ranged between 0.4 and 1.7. Four climatic variables showed distinct relationships with elevation (**Supplementary Figure S1**). Dominant vegetation types included subtropical evergreen forest, temperate deciduous forest and grassland, as well as alpine forest and grassland.

### 2.2 Climate collection and processing

Site-specific elevation values were retrieved from the Shuttle Radar Topography Mission (SRTM) (https://srtm.csi.cgiar.org) dataset at a comparable resolution, based on the recorded geographical coordinates. Climatic variables, including mean annual precipitation (MAP) and mean annual temperature (MAT), were sourced from the WorldClim database at a 30-arcsecond (~1km) spatial resolution (http://www.worldclim.org). For August 2021, vapor pressure deficit (VPD) data were acquired from the TerraClimate repository (Abatzoglou et al., 2018), while the aridity index (AI) was derived from the Global Aridity Index database (Zomer et al., 2022). All data extraction and processing steps were conducted utilizing the “geodata” package in R.

### 2.3 iWUE calculations based on plant carbon stable isotope

The dominant herbs were selected from each site for measuring their foliar stable isotope composition. The mature and intact leaves were selected and dried in an oven at 65 °C to constant weight. The dried leaves were powdered using a grinding mill (Jingxin, JXFSTPRP-32, China), and sieved through a 60-mesh sieve. Foliar δ^13^C and foliar δ^18^O were measured using an isotope ratio mass spectrometer. (Delta V Advantage, Thermo Fisher Scientific, Waltham, MA, USA). The isotope composition of leaves (‰, in parts per thousand) was calculated as (Equation 1):

(1) $$\delta =\left(\frac{R_{sample}}{R_{standard}}-1\right)\times 1000$$

Where *R_sample_* and *R_standard_* are plant leaves stable carbon (*δ*¹³C) and oxygen (*δ*¹^8^O) isotope ratios relative to PDB (Pee Dee Belemnite) standard and SMOW (Standard Mean Ocean Water) standard, respectively. Carbon isotope discrimination (Δ¹³C), representing the isotopic fractionation between plant tissue and atmospheric CO_2_, was computed using the (Equation 2) (Farquhar et al., 1982).

(2) $$\Delta^{13}C=\frac{\left(\delta^{13}C_{a}-\delta^{13}C_{p}\right)}{\left(1+\frac{\delta^{13}C_{p}}{1000}\right)}\;$$

Where *δ¹³C_a_* and *δ¹³C_p_* denote the isotopic composition of atmospheric CO_2_ and the plant sample. *Δ^13^C* additionally integrates a number of individual leaf-level physiological processes (i.e. “photorespiration”, “mesophyll”) (Gong et al., 2022). The concentration of CO_2_ in leaf substomatal cavity (i.e. intercellular CO_2_ [*C_i_*] or chloroplast CO_2_ [*C_c_*]) is calculated by (Equation 3):

(3) $$C_{c}=-\left(C_{a}\times \frac{b-\Delta^{13}C-f^{\prime }\times \frac{\Gamma^{*}}{pC_{a}}}{b-a+\frac{g_{sc}}{g_{m}}\times \left(b-a_{m}\right)}-C_{a}\right)$$

Where the constants *a* (4.4‰), *a_m_* (1.8‰), *b* (29‰), and *f’* (12 ± 4‰) represent the individual fractionations related with the diffusion of CO_2_ in air, across the mesophyll cell, carboxylation by Rubisco, and photorespiratory processes, respectively (Evans and Von Caemmerer, 2013; Gong et al., 2022). *g_sc_/g_m_* (0.79 ± 0.07) represents the ratio of stomatal conductance to CO_2_ to mesophyll conductance to CO_2_ (Ma et al., 2021), and *pC_a_* is the partial pressure of atmospheric CO_2_ determined as a function of *C_a_* and elevation (Stocker et al., 2020). Γ* represents the CO_2_ compensation point in the absence of dark respiration and is calculated by (Equation 4):

(4) $$\Gamma^{*}=\Gamma_{25}^{*}\times \left(\frac{P_{atm\;}}{P_{0}}\right)\times e^{\left(\frac{\Delta H_{a}\times \left(T-298\right)}{RT\times 298}\right)}$$

Where Γ^*^_25_ is the CO_2_ compensation point at 25 °C. *P_atm_* is the atmospheric pressure calculated by a function of elevation (Stocker et al., 2020). *P_0_* is the atmospheric pressure at sea level (101,325 Pa), *ΔH_a_* is the activation energy (37,830 J mol^−1^) (Bernacchi et al., 2001), *T* is the temperature, *R* is the universal gas constant (8.314 J mol^−1^ K) (Mathias and Hudiburg, 2022). The *Ci* can be calculated from *Δ^13^C* further simplified by (Equations 5, 6):

(5) $$C_{i}=\frac{\Delta^{13}C-a-f^{\prime }\times \left(\frac{\Gamma^{*}}{pC_{a}}\right)}{b-a}\times C_{a\;}$$

(6) $$C_{i}=\frac{\Delta^{13}C-a}{b-a}\times C_{a}$$

The corresponding model for C_4_ plants is given by (Equation 7) (Ellsworth and Cousins, 2016):

(7) $$\Delta^{13}{\text{C}}_{4}=a+\left(b4+\left(b3-s\right)\varphi -a\right)\frac{C_{i}}{C_{a}}$$

Here, *b_3_* (30‰) is Rubisco fractionation, *b_4_* (-5.7‰) combines fractionation by phosphoenolpyruvate carboxylase and preceding equilibrium processes, *φ* is bundle-sheath leakiness (set to 0.21 based on representative literature values for grasses under moderate conditions), and *s* (1.8‰) is the fractionation associated with CO_2_ leakage from bundle-sheath cells (Ellsworth and Cousins, 2016; Eggels et al., 2021). Finally, isotope-derived values of *C_i_* (or *C_c_* for the “mesophyll” formulation) are combined with *C_a_* to calculate iWUE (iWUE in μmol CO_2_ mol^−1^ H_2_O) by (Equation 8):

(8) $$iWUE\;=\frac{\left(C_{a}-C_{i}\right)}{1.6}$$

### 2.4 Plant traits and soil biogeochemistry properties

All plant species within each plot were identified. Herbaceous plants were classified taxonomically, collected, and stored in paper envelopes. Aboveground biomass was measured for fresh weight immediately after collection, then oven-dried at 65 °C until constant mass to obtain dry weight. Alpha diversity was assessed using the Shannon-Wiener index and species richness (SR). For each site, species richness was derived from the mean number of species per plot. Shannon-Wiener index and species richness metrics were analyzed with the “vegan” package in R (Dixon, 2003). The dominant herbs were selected from each site for measuring their foliar element content. The community-weighted mean of leaf C, N, and P was determined and weighted by dominant species to represent the community’s average trait value. The dried leaves were powdered using a grinding mill, and sieved through a 60-mesh sieve. Leaf carbon and nitrogen concentrations were quantified with an elemental analyzer (Flash 2000 EA-HT, Thermo Finnigan, Bremen, Germany) (Bai et al., 2025). For leaf phosphorus, samples were digested with H_2_SO_4_–H_2_O_2_ and subsequently analyzed via the molybdate/stannous chloride colorimetric procedure, using an automated discrete analyzer (Li et al., 2025) (DeChem-Tech GmbH Inc., Hamburg, Germany).

At each site, three replicate topsoil cores (0–10 cm depth) were obtained with a 7 cm diameter auger, then placed in sealed plastic bags, and transported to the laboratory. The collected soil was air-dried, passed through a 2 mm mesh sieve to homogenize the soil samples, and any visible roots or stone fragments were manually removed prior to analysis. For the determination of soil organic carbon (SOC) and soil total nitrogen (STN), inorganic carbonates were first eliminated by treating the samples with 1 N HCl for 24 hours, after which measurements were performed with an elemental analyzer (Vario EL, Elementar Analysensysteme GmbH, Langenselbold, Germany). Soil bulk density was assessed from intact cores of 5 cm diameter.

### 2.5 Statistical analysis

The effects of elevation on grasses iWUE were evaluated using linear mixed-effects models (LMMs), treating the identity of the dominant species as a random effect to account for interspecific variation. Parameter estimation for these models was performed via restricted maximum likelihood (REML) with the “lmer” function from the “lme4” R package (Bates et al., 2015). To determine the relative contribution of different factors to iWUE variation, we used the Extreme Gradient Boosting (XGBoost) and Shapley Additive Explanation (SHAP) models to analyze the influence of multi-variables. XGBoost, a machine learning algorithm, is designed to reduce overfitting risks without compromising its ability to capture intricate nonlinear associations (Sheridan et al., 2016). SHAP (SHapley Additive exPlanations), which originates from cooperative game theory’s Shapley value, offers a consistent framework for interpreting predictions from diverse machine learning models and visualizing the complex interactions between the dependent variable and its factors (Lundberg et al., 2020; Ma et al., 2025). Before model fitting, we tested for the multicollinearity among independent variables by calculating the variance inflation factor (VIF) values for each variable, and only included factors with VIF < 10 as model inputs (Yan et al., 2024). The relative importance of factors was calculated the mean of absolute SHAP values. The analysis was implemented with the “xgboost” and “shapviz” packages in R.

## 3 Results

### 3.1 Elevational patterns of plant carbon and oxygen stable isotope and iWUE

**Figure 2:**
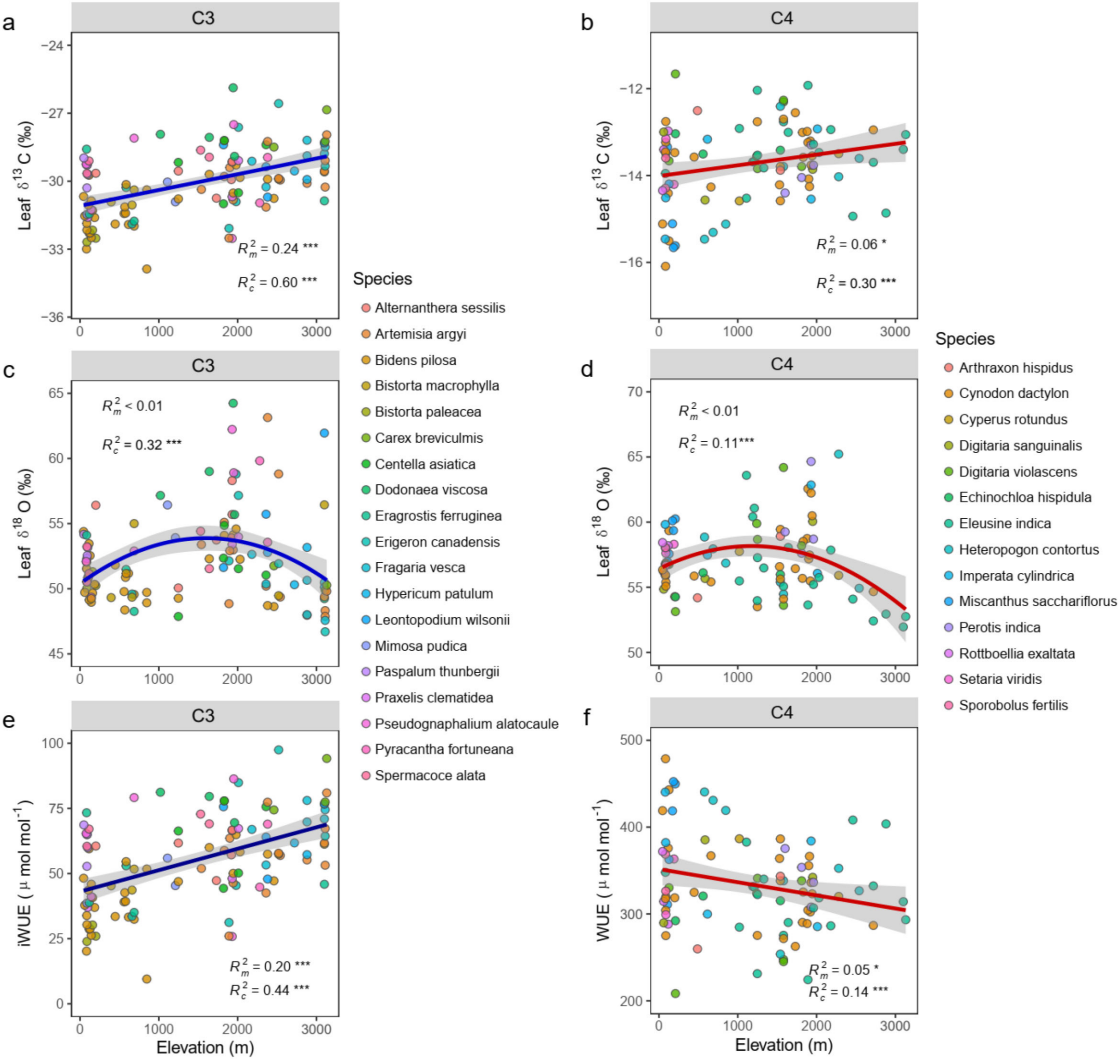
Relationships of the leaf δ^13^C **(a, b)**, leaf δ^18^O **(c, d)**, iWUE **(e, f)** for C_3_ grasses (left) and C_4_ grasses (right) with elevation. Colored points correspond to values with different dominant species and lines represent the overall coefficients across all species from linear mixed-effects models (LMMs). Shaded areas denote 95% confidence intervals and significance levels are as follows: *p < 0.05, **p < 0.01, and ***p <0.001. The marginal (R^2^_m_) and conditional (R^2^_c_) R-squared represent fractions of variance explained by fixed effects (elevation) and fixed-random effects (elevation + species), respectively.

Across the transect, the mean leaf δ^13^C values of C_3_ (-30.07 ‰) plant were significantly more depletion than δ^13^C values of C_4_ grasses(-13.72 ‰)(p<0.001, **Supplementary Figure S2**), but exhibited a similar upward trend with elevation rising (C_3_: R^2^_C_ =0.60, p<0.001, C_4_: R^2^_C_ =0.30, p<0.001, **Figure 2**). Along the elevation gradient, leaf δ^13^C values rose by approximately 2‰ per 1000 m. Interestingly, a hump-shaped relationship was observed between leaf δ^18^O and elevation for both photosynthetic types (C_3_: R²_c_ = 0.32, p< 0.001; C_4_: R²_c_ = 0.11, p< 0.001, **Figure 2**). Unexpectedly, we found that the iWUE of C_3_ grasses increased significantly with elevation (R²_m_ = 0.20 p< 0.001), whereas a slight but significant decrease was observed in C_4_ grasses (R²_m_ = 0.05, p< 0.05; **Figure 2**). Incorporating species composition as a random factor substantially improved the model’s explanatory power for iWUE variation along the gradient (C_3_: R²_c_ = 0.44 C_4_: R²_c_ = 0.14; both p< 0.001). We also found the similar relationships between the iWUE and leaf δ^18^O both in C_3_ and C_4_ grasses, iWUE was positively correlated with leaf δ^18^O in both C_3_ (R² = 0.12, p< 0.001) and C_4_ grasses (R² = 0.09, p = 0.003; **Supplementary Figure S3**).

### 3.2 Factors determining the elevational changes in iWUE

**Figure 3:**
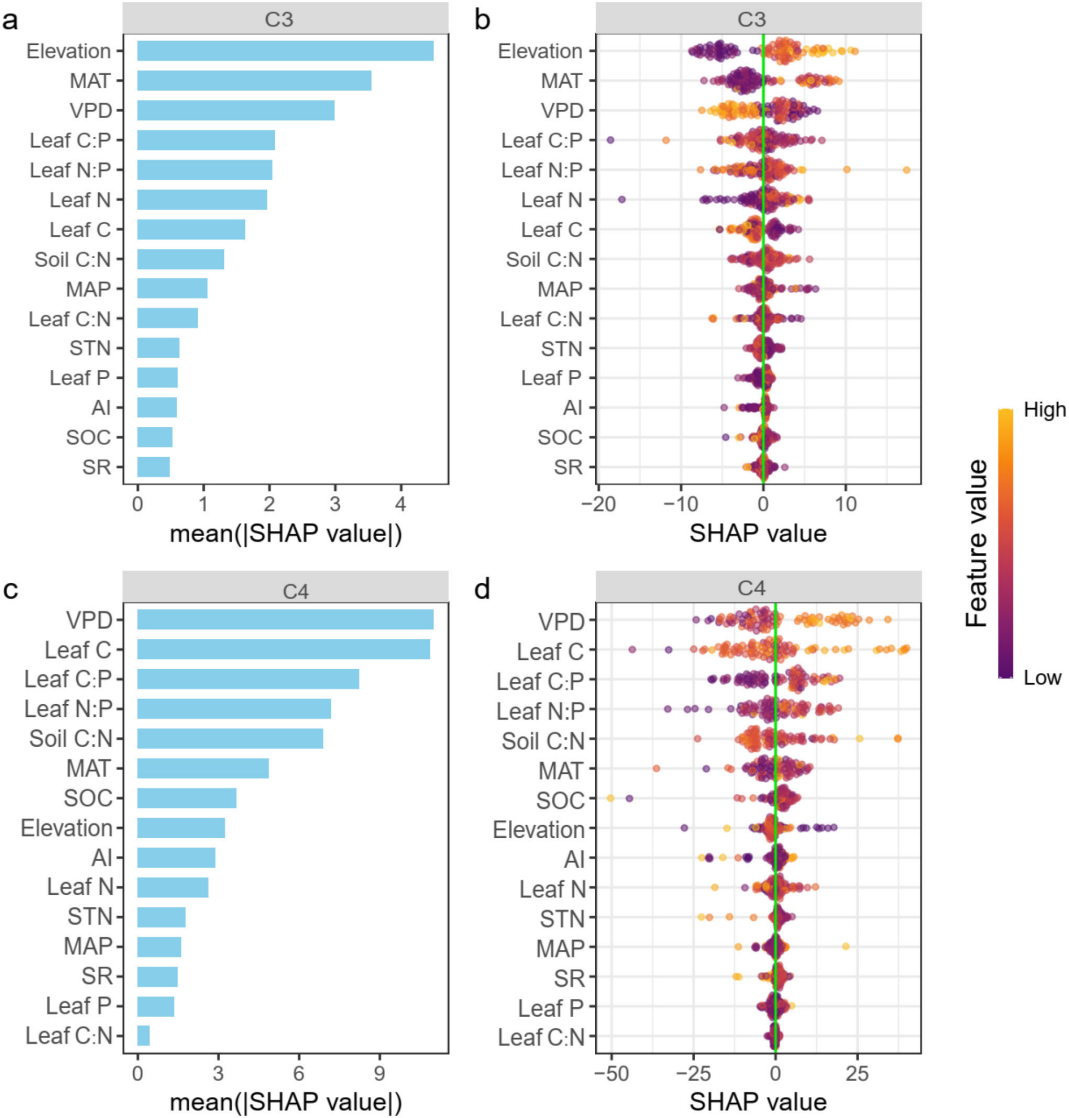
The relative importance of the multi-factors on iWUE of C3 **(a, b)** and C4 grasses **(c, d)**. The factors are sorted by feature importance calculated by averaging the absolute Shapley values of a given factor. Each dot on the plot is a Shapley value for a given factor and observation. The color gradient of dots from blue to yellow indicate low to high feature values, respectively. The Shapley values greater than 0 represent positive effects, while the values less than 0 indicate negative effects. MAT, mean annual temperature; MAP, mean annual precipitation; VPD, vapor pressure deficit; AI, aridity index; SR, Species Richness; Leaf C, leaf C content; Leaf P, leaf P content; Leaf N, leaf N content; Leaf C:N, leaf C:N ratio; Leaf N:P, leaf N:P ratio; Leaf C:P, leaf C:P ratio; STN, soil total nitrogen; SOC, soil organic carbon; Soil C:N, soil C:N ratio.

**Figure 4:**
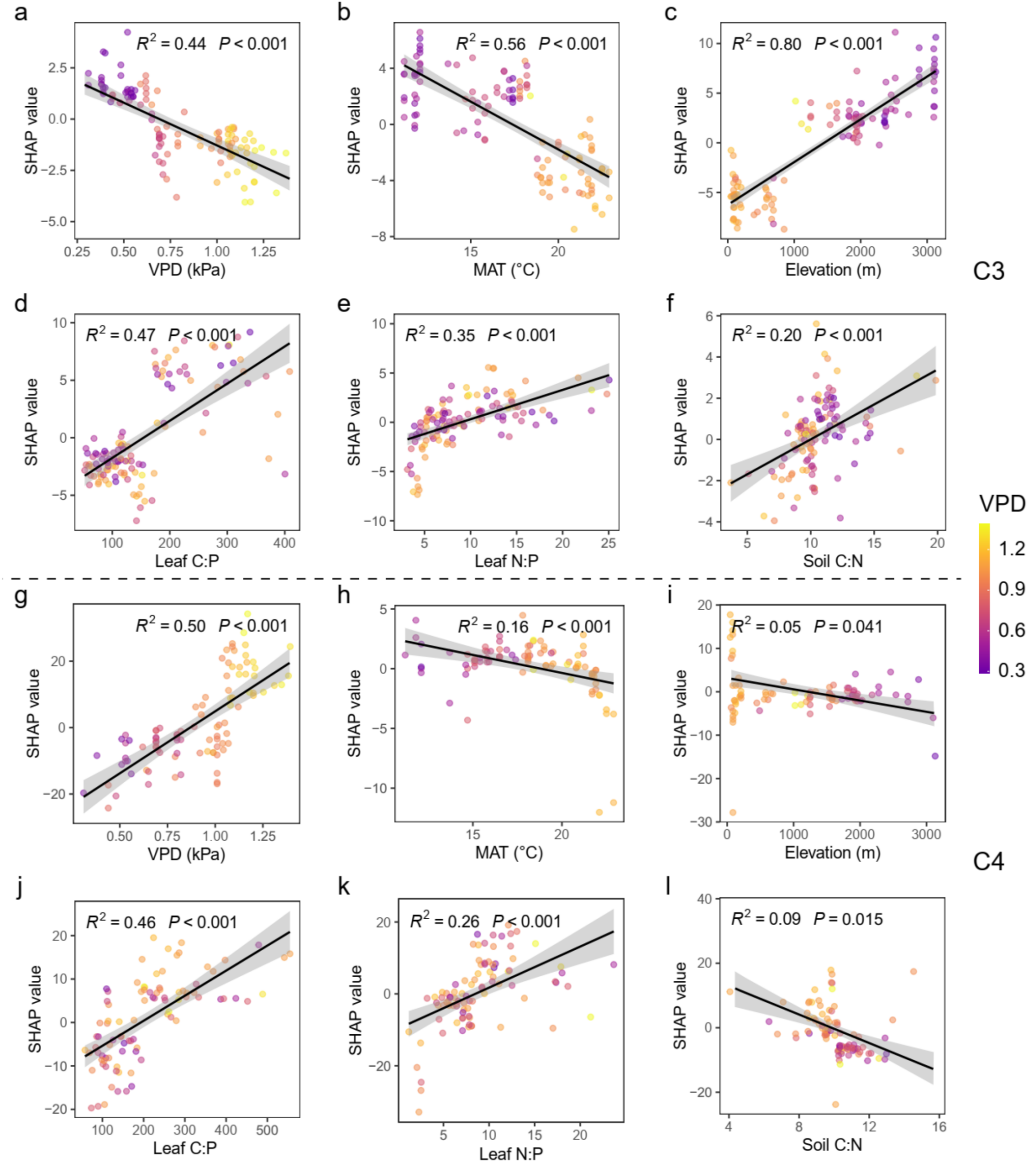
Shapley feature dependence plots based on the extreme gradient boosting (XGBoost) model showing the relationships between the selected variables and iWUE of C3 **(a–f)** and C4 **(g–l)** grasses. The color gradient of dots from blue to yellow indicate low to high values of VPD, respectively. MAT, mean annual temperature; VPD, vapor pressure deficit; Leaf N:P, leaf N:P ratio; Leaf C:P, leaf C:P ratio; Soil C:N, soil C:N ratio.

We employed the SHAP algorithm within the XGBoost model to quantified the relative contribution of each factor to the changes of iWUE. For C_3_ grasses, elevation, MAT, VPD were the three most important factors to account for the changes in iWUE (**Figure 3**). When it comes to C_4_ grasses, the top three most significant factors were VPD, leaf C content, and leaf C:P ratio (**Figure 3**). Specifically, VPD is negatively correlated with the iWUE of C_3_ grasses (R² = 0.44, p< 0.001), but positively related to the iWUE of C_4_ grasses (R² = 0.50, p< 0.001) (**Figure 4**). The iWUE in C_3_ and C_4_ was also conversely respond to elevation rising. However, the MAT exhibits a negative correlation with the iWUE both in C_3_ and C_4_ grasses. Furthermore, the effects of MAT and elevation on iWUE in C_3_ grasses (MAT, R² = 0.56, p< 0.001, Elevation, R² = 0.80, p< 0.001) were stronger than those in C_4_ grasses (MAT, R² = 0.16, p< 0.001, Elevation, R² = 0.05, p = 0.041) (**Figure 4**). The stoichiometric traits where the second most important factors explain the changes in iWUE both for C_3_ and C_4_ grasses. Generally, the leaf N:P ratio and leaf C:P exerted a positive effect on the changes of iWUE both for C_3_ (leaf N:P, R² = 0.35, p< 0.001, leaf C:P, R² = 0.47, p< 0.001) and C_4_ (leaf N:P, R² = 0.26, p< 0.001, leaf C:P, R² = 0.46, p< 0.001) grasses. However, the iWUE of C_3_ and C_4_ grasses was inversely respond to soil C:N ratio. The iWUE of C_3_ was positively corelated the soil C:N ratio, while exhibit a negative relationship between iWUE and soil C:N ratio in C_4_ grasses.

## 4 Discussion

Our study reveals the altitude-adaptive patterns of iWUE between C_3_ and C_4_ grasses along an elevational transect. While iWUE increased significantly with elevation for C_3_ species, it exhibited a slight but significant decrease for C_4_ species (**Figure 2**). This core finding, derived from stable carbon (δ¹³C) and oxygen (δ¹^8^O) isotopes, highlights the distinct eco-physiological strategies and mechanisms that how plants adapt to co-varying environmental gradients. The application of a machine learning framework (XGBoost-SHAP) identified climate variables (VPD, MAT), and plant-soil stoichiometric traits (leaf N:P, leaf C:P, soil C:N) as the key drivers of these adaptive patterns (**Figures 3** and **4**). Notably, the direction and magnitude of the effects exerted by these drivers differed substantially between the two functional plant types.

The opposing iWUE responses to elevation are rooted in the fundamental physiological differences between C_3_ and C_4_ photosynthesis pathway. The C_4_ pathway, with its CO_2_-concentrating mechanism (CCM), confers an inherent advantage in water-use efficiency by enabling high rates of carbon fixation at lower stomatal conductance (*g_s_*) (Ehleringer and Cerling, 2001; Way et al., 2014). This is consistently reflected in our data by the significantly less negative (enriched) leaf δ¹³C values in C_4_ grasses along the elevational gradient (**Figure 2**). The classical model of carbon isotope discrimination (Δ) suggests that leaf δ¹³C is related to the ratio of intercellular to atmospheric CO_2_ concentration (c_i_/c_a_), which is inversely correlated with iWUE (Farquhar et al., 1982). For C_3_ plants, a reduction in c_i_/c_a_ directly leads to higher iWUE. This pattern is commonly observed in mountain ecosystems and is often attributed to stomatal limitation under cooler temperatures (Körner, 2007; Maxwell et al., 2018). In contrast, the slight decline in C_4_ iWUE with increasing elevation is interesting. It suggests that the C_4_ advantage is constrained under the cold conditions of high altitudes (**Figure 2**). At higher elevations, lower temperatures can inhibit the activity of key C_4_ enzymes like PEP carboxylase, which is crucial for initial CO_2_ fixation (Carvalho et al., 2024). This reduced enzymatic efficiency weakens the CO_2_ concentrating rate, potentially lowering water use efficiency (Carvalho et al., 2024). Meanwhile, cold stress may disrupt the structural integrity of the specialized Kranz anatomy. It can also weaken the efficiency of metabolite shuttling between mesophyll and bundle sheath cells (Sage et al., 2011). These combined biochemical and anatomical constraints explain the observed decline in C_4_ plant WUE along elevation gradients (Sage and Kubien, 2007; Way et al., 2014). Consequently, while stomatal conductance may still decrease with elevation (as suggested by rising δ¹³C), the potential parallel decline in photosynthetic rate might be proportionally greater, leading to a net decrease in iWUE. This finding highlights that the higher iWUE of C_4_ plants is context-dependent and can be diminished by environmental factors. However, it should be noted that this study used the fixed φ value to calculate the iWUE of C_4_ plants. This simplification may introduce a systematic bias in absolute iWUE estimates across the elevation gradient. Nevertheless, the fixed φ value represents a necessary compromise, as deriving species- and site-specific leakiness under field conditions remains unfeasible. Additionally, φ variation in response to environmental drivers (e.g., light and temperature) is often limited to moderate ranges (Kromdijk et al., 2016). Consequently, the relative C_4_ iWUE trends and their ecological interpretation are likely robust, though absolute values should be interpreted with appropriate caution.

Our analysis identified VPD as a key climatic driver for both photosynthesis pathways, but with diametrically opposite effects: a strong negative correlation with iWUE in C_3_ grasses and a strong positive correlation in C_4_ grasses (**Figures 3** and **4**). This contrast highlights differential stomatal regulation strategies along the elevation rising. For C_3_ plants, high VPD primarily triggers stomatal closure to prevent water loss (Wang et al., 2012; Li et al., 2024). However, long-term water stress can lead to significant non-stomatal limitations, such as reduced mesophyll conductance (the ease of CO_2_ diffusion to chloroplasts) and impaired biochemical capacity for carboxylation, which further constrain photosynthesis independently of stomatal aperture (Gimeno et al., 2019; Chen et al., 2025). This aligns with observations in some arid systems where iWUE increases or declines under severe drought stress (Cooley et al., 2022; Huang and Zhai, 2023). For C_4_ plants, their CO_2_-concentrating mechanism (CCM) provides a critical advantage (Zait et al., 2024). It actively elevates CO_2_ concentration around the enzyme Rubisco. Therefore, when stomata close in response to high VPD to conserve water, the internal CO_2_ reservoir helps sustain photosynthetic rates for longer periods compared to C_3_ plants (Márquez et al., 2024). Notably, recent evidence shows that C_4_ plants also employ non-stomatal control under VPD stress, which further limits water loss while helping maintain a favorable CO_2_ fixation (Gan and Sage, 2024; Márquez et al., 2024). In summary, under high VPD, C_3_ plants face a challenge where stomatal closure is rapidly followed by non-stomatal metabolic limitations. In contrast, C_4_ plants are more resilient; their CCM buffers the carbon supply against stomatal closure.

**Figure 5:**
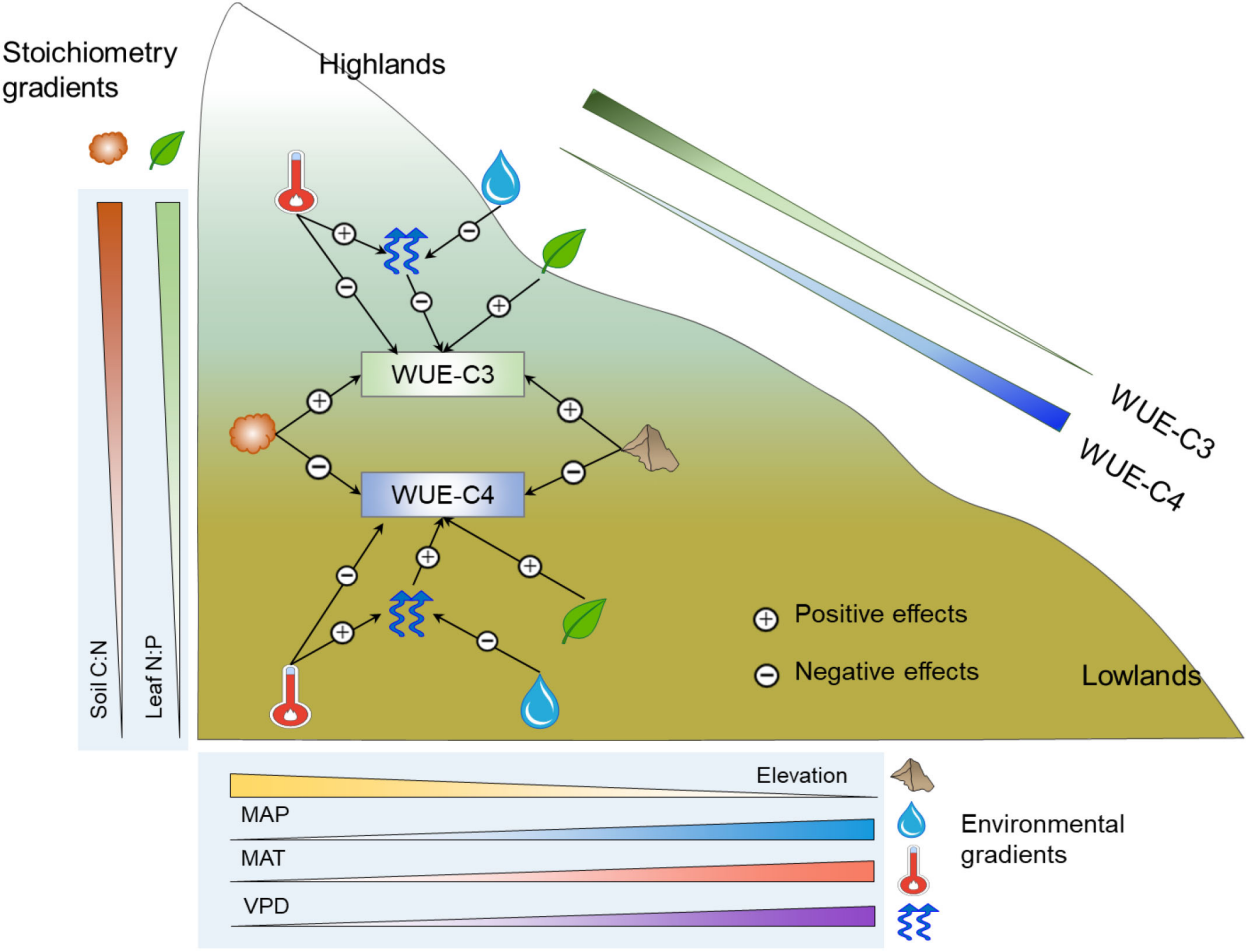
The conceptual diagram for the multivariate effects of climatic gradients, soil properties, and plant stoichiometries on iWUE along the elevation. Plus symbols indicate a positive effect to iWUE, whereas minus symbols indicate a negative effect.

The prominent role of plant and soil stoichiometry highlights the tight coupling between plant water-use strategies and nutrient economics (Wright et al., 2004; Du et al., 2021). The positive relationships between iWUE and leaf C:P and N:P ratios in both pathways suggest a coordinated shift towards a more conservative resource-use strategy. High leaf C:P ratios are indicative of greater investment in structural carbon (e.g., cell walls, fibers) or storage compounds relative to metabolically active nutrient pools (e.g., phosphorus) (Xing et al., 2021). This is consistent with the “Leaf Economics Spectrum,” where species characterized by slow growth rates, longer leaf lifespans, and higher tissue density typically exhibit elevated iWUE (Reich, 2014; Luo et al., 2018). The positive effect of N:P ratio further implies that phosphorus limitation, or a shift towards a more higher P-efficient use traits (e.g., thicker leaves, lower *g_s_*) that enhance iWUE (Pinto et al., 2016; Wang and Wen, 2022). P limitation can directly constrain photosynthetic metabolism by limiting ATP synthesis and RuBP regeneration, which drives a coordination between nutrient use and water use strategies (Kumar et al., 2021; Odoom and Ofosu, 2024). When non-stomatal limitations (e.g.,leaf anatomy or biochemistry) intensify due to phosphorus scarcity, plants may strategically reduce stomatal conductance to conserve water—given the limited potential for additional carbon gain (Dijkstra et al., 2016). In terms of nitrogen limitation, C_4_ plants generally have higher nitrogen use efficiency compared to C_3_ plants (Bai et al., 2025). Under lower N conditions, the priority of C_4_ plants tend be to allocate the limited N to maintain the complex CCM unit (e.g., bundle sheath structure) and associated high metabolic rates (Ghannoum et al., 2011). This allocation might come at the cost of optimal stomatal control or leaf hydraulic architecture, potentially reducing iWUE (Togawa and Ueno, 2022). Furthermore, the opposite responses of C_3_ and C_4_ iWUE to soil C:N ratio is a compelling result (**Figures 4** and **5**). For C_3_ grasses, the positive correlation between iWUE and soil C:N ratio indicates that plants adopt a conservative strategy under low nitrogen availability (high soil C:N) (**Figure 4**). Specifically, they tend to invest in long-lived tissues with slow gas exchange, which inherently leads to higher iWUE (**Figure 4**). This divergence underscores that the integration of nutrient and water economies is pathway-specific.

These findings have significant implications for predicting grassland responses to global change. Under a warming climate (increased MAT and VPD), low-elevation C_3_ grasses may face intensified environmental pressure due to decreased iWUE. C_4_ grasses might expand their advantage in areas where rising VPD is the dominant change, but their performance will be modulated by temperature and nutrient dynamics. Furthermore, atmospheric nitrogen deposition, which reduces soil C:N, may exert differential effects on grassland community composition. Specifically, it could favor C_3_ grasses by mitigating the limitation imposed by decreased iWUE or benefit C_4_ grasses by alleviating constraints on their iWUE, with the specific outcome dependent on environmental contexts. Future research should aim to mechanistically link the stoichiometric signals to key specific leaf traits (e.g., leaf mass per area, hydraulic conductance) and microbial processes governing nutrient cycling. Integrating these relationships into dynamic vegetation models will improve forecasts of ecosystem carbon-water fluxes in montane grasslands under changing climate.

## 5 Conclusions

This study demonstrates a fundamental divergence in how C_3_ and C_4_ grasses regulate their iWUE along an elevational gradient. We found that iWUE increased with elevation for C_3_ species but slightly decreased for C_4_ species. These contrasting patterns are driven by the integrated effects of climate and plant-soil stoichiometry, operating through pathway-specific mechanisms. The opposing responses of iWUE to VPD—negative for C_3_ and positive for C_4_ grasses—and to soil C:N ratio, highlighting distinct strategies in coupling carbon, water, and nutrient economies. The results highlight that the iWUE of C_4_ plants is context-dependent, which may be constrained by lower temperatures in high-elevation environments. In contrast, C_3_ plants enhance their iWUE under the cooler and nutrient-limited conditions with a conservative resource-use strategies. This research advances our understanding of plant adaptive strategies by integrating stable isotope ecology with stoichiometric theory, revealing that iWUE patterns are not simply climate-driven but emerge from multi-factor interactions specific to photosynthetic pathway. The findings have critical implications for predicting shifts in grassland community structure and ecosystem function under future climate change, particularly where rising VPD, global warming, nitrogen deposition. Integrating these relationships into dynamic vegetation models will improve forecasts of ecosystem carbon-water fluxes in montane grasslands under changing climates.

## Data Availability

The datasets presented in this study can be found in online repositories. The names of the repository/repositories and accession number(s) can be found below: https://doi.org/10.5061/dryad.4mw6m90p5.

## Supplementary material

Supplementary Figure 1 The relationships of the mean annual temperature (MAT, **a**), mean annual precipitation (MAP, **b**), aridity index (AI, **c**), vapor pressure deficit (VPD, **d**) and with elevation. GX, Guanxi province, YN, Yunnan province. All, overall trend for all data.

Supplementary Figure 2 Density plot showing the data distribution of leaf δ^13^C **(a)** and leaf δ^18^O **(c)**, vertical dashed lines indicate group means value. One-way ANOVA comparisons of leaf δ^13^C **(b)** and leaf δ^18^O **(d)** in C_3_ and C_4_ grass. The significance levels are as follows: *p< 0.05; **p< 0.01; ***p< 0.001.

Supplementary Figure 3 Relationships of the iWUE at C_3_ grasses **(a)** and C_4_ grasses **(b)** with leaf δ^18^O. The solid fitted lines are significant at p< 0.05, while the grey shadow areas indicate the 95% confidence interval for the fitted line.
