# Supplementary material for: Altitude-adaptive water use strategies of grassland are constrained by air dryness and stoichiometry in southwest of China
Source: Front Plant Sci. 2026 Feb 9;17:1773262. doi: 10.3389/fpls.2026.1773262 (PMC12926462; doi:10.3389/fpls.2026.1773262)
Supplement: Supplementary Figure 1 — The relationships of the mean annual temperature (MAT, a), mean annual precipitation (MAP, b), aridity index (AI, c), vapor pressure deficit (VPD, d) and with elevation. GX, Guanxi province, YN, Yunnan province. All, overall trend for all data. [file SupplementaryFile1.docx]

**Supplementary materials**

**Figure S1-S3**


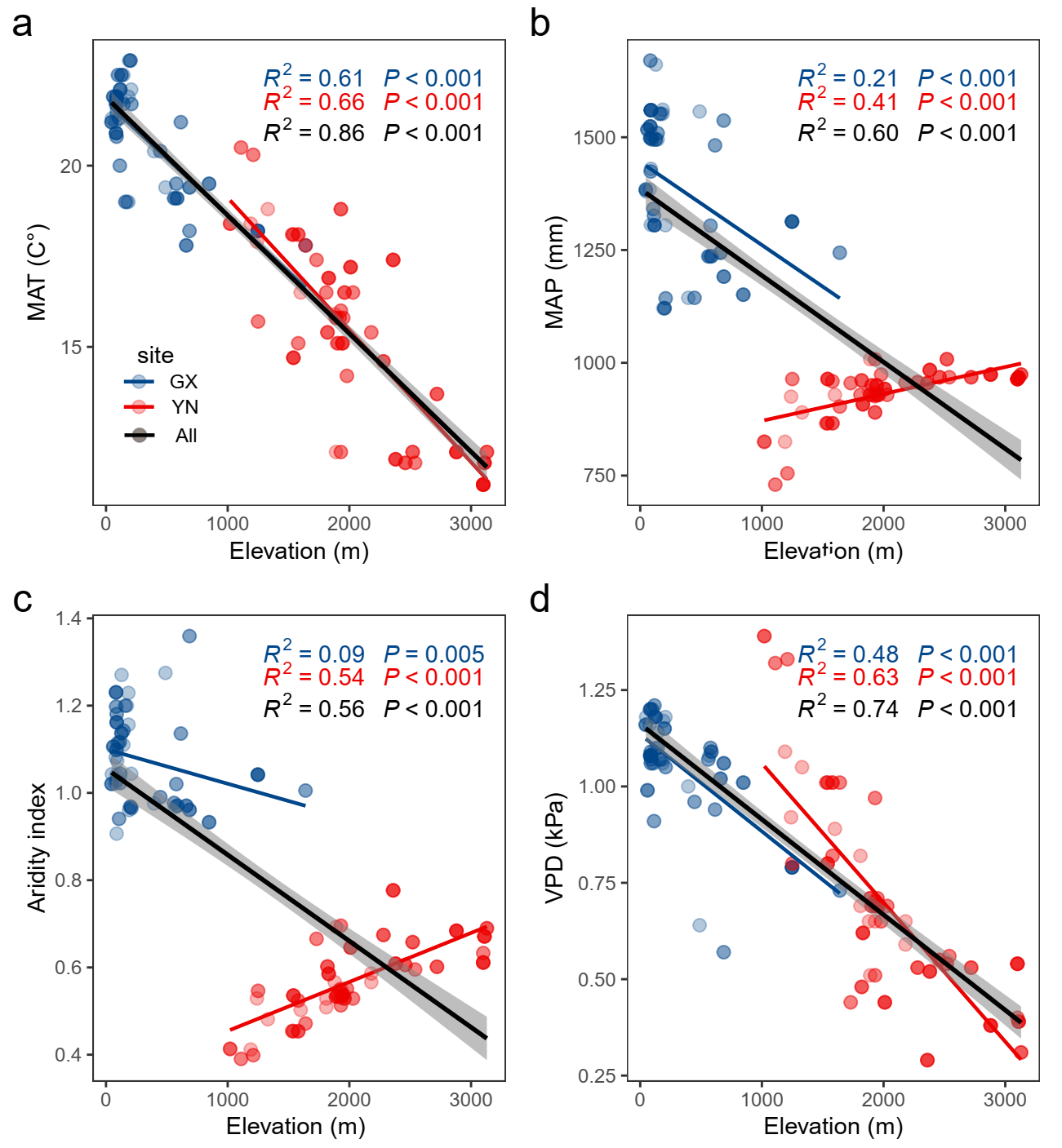


**Figure. S1** The relationships of the mean annual temperature (MAT, a), mean annual precipitation (MAP, b), aridity index (AI, c), vapor pressure deficit (VPD, d) and with elevation. GX, Guanxi province, YN, Yunnan province. All, overall trend for all data.


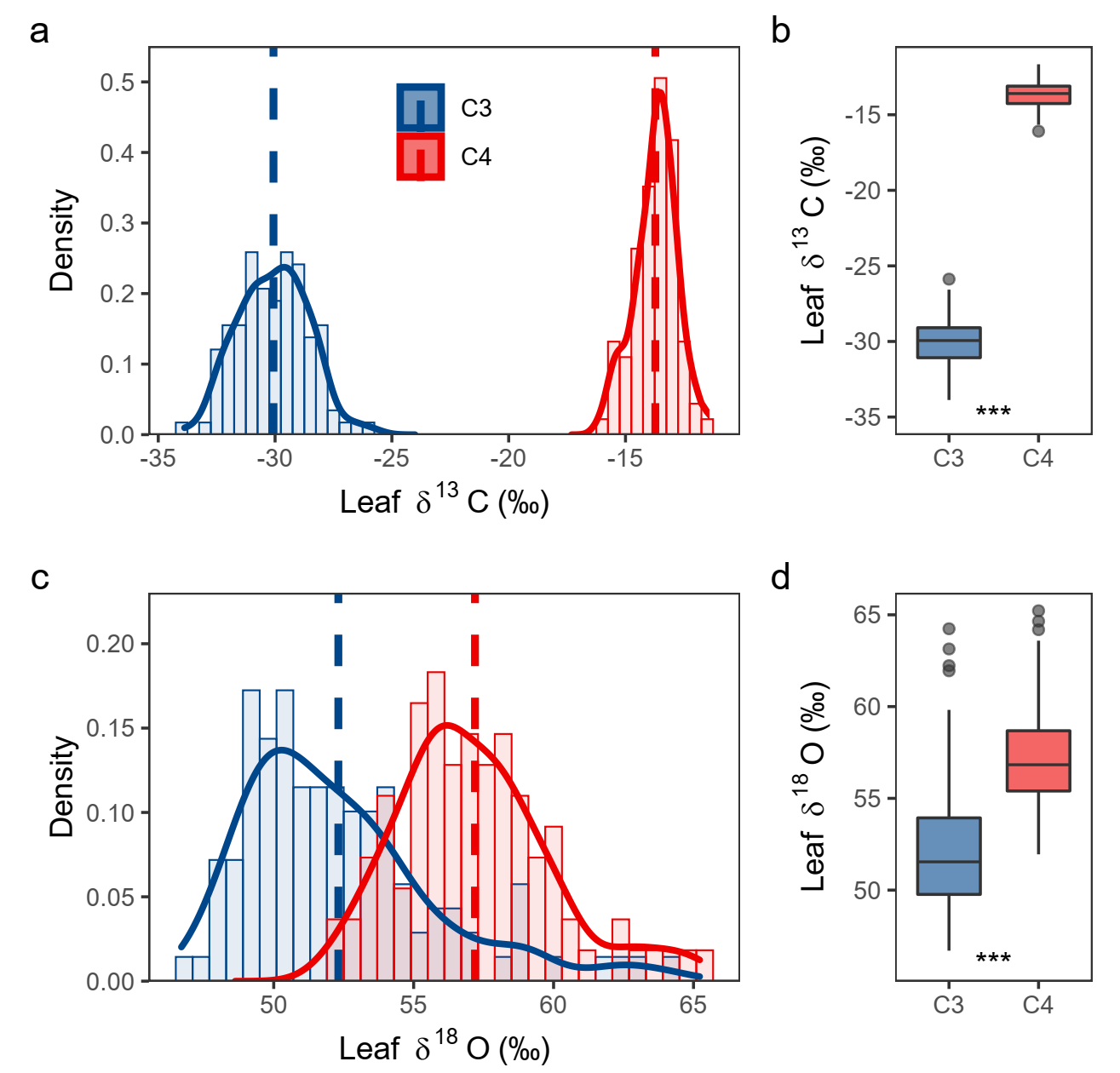


**Figure.S2** Density plot showing the data distribution of leaf δ^13^C (a) and leaf δ^18^O (c), vertical dashed lines indicate group means value. One-way ANOVA comparisons of leaf δ^13^C (b) and leaf δ^18^O (d) in C_3_ and C_4_ grass. The significance levels are as follows: *p< 0.05; **p < 0.01; ***p < 0.001.


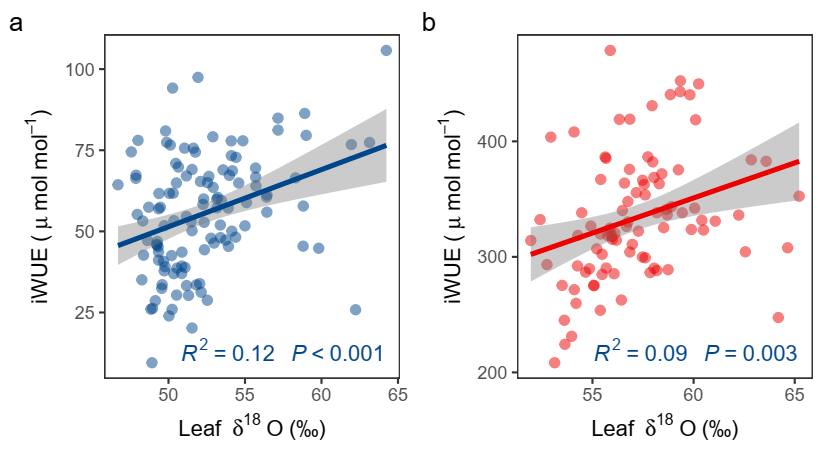


**Figure.S3** Relationships of the iWUE at C_3_ grasses (a) and C_4_ grasses (b) with leaf δ^18^O. The solid fitted lines are significant at p< 0.05, while the grey shadow areas indicate the 95% confidence interval for the fitted line.
